# Patient-Derived Ex Vivo Cultures and Endpoint Assays with Surrogate Biomarkers in Functional Testing for Prediction of Therapeutic Response

**DOI:** 10.3390/cancers15164104

**Published:** 2023-08-15

**Authors:** Yoshiyuki Tsukamoto, Yuka Hirashita, Tomotaka Shibata, Shoichi Fumoto, Shusaku Kurogi, Chisato Nakada, Keisuke Kinoshita, Takafumi Fuchino, Kazunari Murakami, Masafumi Inomata, Masatsugu Moriyama, Naoki Hijiya

**Affiliations:** 1 Department of Molecular Pathology, Faculty of Medicine, Oita University, 1-1 Hasama-machi, Oita 879-5593, Japan; 2 Department of Gastroenterology, Faculty of Medicine, Oita University, Oita 879-5593, Japan; 3 Department of Gastroenterological and Pediatric Surgery, Faculty of Medicine, Oita University, Oita 879-5593, Japan; 4 Department of Surgery, Oita Nakamura Hospital, Oita 879-5593, Japan; 5 Department of Urology, Faculty of Medicine, Oita University, Oita 879-5593, Japan

**Keywords:** functional testing, patient derived cancer organoids (PDCOs), patient derived explants (PDEs), 3-(4,5-Dimethylthiazol-2-yl)-2,5-diphenyltetrazolium bromide (MTT), lactate dehydrogenase (LDH), spheroid, collagen droplet-embedded culture drug sensitivity test (CD-DST), extracellular matrix (ECM), organs on chips, micro-organospheres (MOSs)

## Abstract

**Simple Summary:**

Studies aimed at prediction of chemotherapeutic efficacy using patient-derived ex vivo cultures (referred to here as “functional testing”) have been increasing. The present review provides information on the various types of ex vivo cultures and endpoint assays that employ a range of surrogate biomarkers of drug response. As ex vivo cultures for functional testing, two-dimensional cultures, spheroids, organoids, explants (including histoculture), microfluid-based culture, and micro-organospheres are introduced. The endpoint assays described include ATP-based bulk assay, dynamic BH3 profiling, optical metabolic imaging, fluorescence lifetime imaging microscopy, fluorescent dye-based assay, mass accumulation rate assay, live cell imaging-based assay, and immunostaining for drug-specific response biomarkers. The advantages and disadvantages of these culture systems and endpoint assays are discussed.

**Abstract:**

Prediction of therapeutic outcomes is important for cancer patients in order to reduce side effects and improve the efficacy of anti-cancer drugs. Currently, the most widely accepted method for predicting the efficacy of anti-cancer drugs is gene panel testing based on next-generation sequencing. However, gene panel testing has several limitations. For example, only 10% of cancer patients are estimated to have druggable mutations, even if whole-exome sequencing is applied. Additionally, even if optimal drugs are selected, a significant proportion of patients derive no benefit from the indicated drug treatment. Furthermore, most of the anti-cancer drugs selected by gene panel testing are molecularly targeted drugs, and the efficacies of cytotoxic drugs remain difficult to predict. Apart from gene panel testing, attempts to predict chemotherapeutic efficacy using ex vivo cultures from cancer patients have been increasing. Several groups have retrospectively demonstrated correlations between ex vivo drug sensitivity and clinical outcome. For ex vivo culture, surgically resected tumor tissue is the most abundant source. However, patients with recurrent or metastatic tumors do not usually undergo surgery, and chemotherapy may be the only option for those with inoperable tumors. Therefore, predictive methods using small amounts of cancer tissue from diagnostic materials such as endoscopic, fine-needle aspirates, needle cores and liquid biopsies are needed. To achieve this, various types of ex vivo culture and endpoint assays using effective surrogate biomarkers of drug sensitivity have recently been developed. Here, we review the variety of ex vivo cultures and endpoint assays currently available.

## 1. Introduction

Drug efficacy varies widely among individuals with cancer. For example, neoadjuvant chemotherapy (NAC) with docetaxel, cisplatin, and 5-fluorouracil (DCF) is recommended for locally advanced esophageal squamous cell carcinoma [1]. Even with such a potent combination of cytotoxic drugs, the proportion of patients who achieve complete response is only 30% [2,3], and patients who have residual cancer after DCF-NAC have a higher recurrence rate and an unfavorable prognosis. To reduce side effects and improve the efficacy of chemotherapy, prediction of therapeutic outcomes in cancer patients is important. Currently, the most widespread method for predicting the efficacy of anti-cancer drugs is gene panel testing using next-generation sequencing (NGS). Increases in the number of panel genes and the development of artificial intelligence (AI) methods are expected to improve the accuracy of this approach. However, Pauli et al. have estimated that even if whole-exon sequencing is performed, the optimal anti-cancer drug will be selected for only 10% of patients [4]. In fact, in Japan, between September 2019 and August 2020, only 8.1% of diagnosed patients received an optimal selected anti-cancer drug (Report of the Roundtable Consortium on the Promotion of Cancer Genomic Medicine in Japan). Additionally, even if optimal drugs can be selected, a significant proportion of patients do not benefit from the indicated therapy [5]. In addition, most of the anti-cancer drugs selected using gene panel testing are molecularly targeted drugs, and it remains difficult to predict the efficacy of cytotoxic drugs, such as fluoropyrimidines, platinum drugs and taxanes, which are still used as standard therapy for many cancers, especially those of the digestive tract. Therefore, panel testing alone is not sufficient for personalized medicine.

In parallel with gene panel testing, methods for predicting the efficacy of anti-cancer drugs using ex vivo culture of cancer tissue from patients and direct application of anti-cancer drugs to observe the response (referred to here as “functional testing”) have long been underway. Functional testing is designed to select the optimal anti-cancer drugs for individual patients by testing the ex vivo sensitivity of patient-derived cancer cells to those drugs. In this review, the concept of ex vivo culture is expanded to include various types of ex vivo culture of patient-derived cancer cells, such as two-dimensional (2D) culture, 3D spheroids, 3D organoids, explants, and microfluidic-based culture.

Functional testing using patient-derived ex vivo cultures began in the 1970s using agar with a feeder layer [6]. Several papers concluded that such functional testing was not recommended for clinical diagnostics because most investigations of chemotherapy sensitivity and resistance assays (CSRAs), which we have included within the category of functional testing here, did not meet the criteria that had been established by the ASCO-related Working Group [7,8,9]. In Japan, however, a number of institutions have conducted CSRAs using various methods, including the succinic dehydrogenase inhibition method (SDI), collagen droplet-drug sensitivity test (CD-DST), and histoculture drug response assay (HDRA), and investigated the correlation between ex vivo drug sensitivity and clinical response. Kondo et al. summarized the results of CSRAs in 1101 patients at 42 institutions [10]; the true positivity rate is 46.4%, the true negativity rate 93%, and the accuracy 73.6%. Based on these cumulative data for CSRAs in Japan, two types of functional tests—CD-DST and HDRA—were approved for advanced medical care around 2007 and have been covered by health insurance since 2012. In addition to these assays, recent advances in ex vivo culture technology, such as organoid culture and explants, have accelerated functional testing and a number of studies have demonstrated that ex vivo sensitivity correlates with patient therapeutic efficacy.

**Figure 1:**
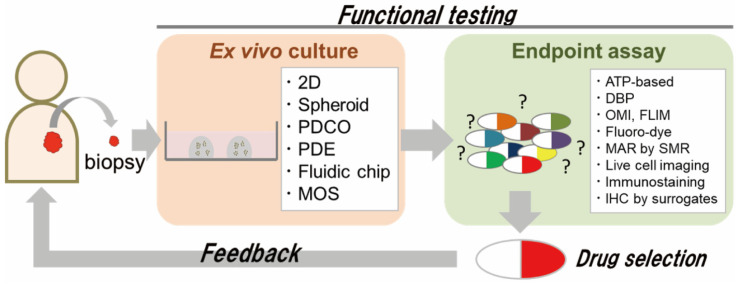
Scheme of functional testing for prediction of clinical response. Functional testing involves a combination of ex vivo culture and endpoint assay. For rapid and effective functional testing, careful choice of methods from the two categories is important.

Providing feedback on the diagnostic results of ex vivo functional testing to patients, i.e., to achieve clinical translation of functional testing, presents two major challenges. One is ex vivo culture using small amounts of patient cancer tissue, such as diagnostic biopsy samples (Figure 1). In patients with inoperable cancers, such as metastases or recurrences, sufficient cancer tissue cannot be obtained, and chemotherapy is the mainstay of their treatment. In addition, neoadjuvant chemotherapy has recently become the standard of care for many patients with solid tumors. Neoadjuvant chemotherapy is designed to reduce the size of cancer before surgery or to eliminate a fraction of the cancer cells that have disseminated from the primary tumor in patients with early metastatic carcinomas (e.g., a proportion of those with esophageal or pancreatic cancer). Most neoadjuvant chemotherapies involve potent cytotoxic drugs, but nevertheless, certain patients do not respond. In such patients, the duration of neoadjuvant chemotherapy (about 3 months for esophageal cancer) leads to unnecessary cancer growth. Therefore, if ex vivo cultures could be established from small amounts of cancer tissue for testing purposes, more patients would be eligible.

The other challenge is the method used for endpoint assays in functional testing (Figure 1). When preparing ex vivo cultures from small amounts of cancer tissue, it is necessary to expand the cultures, which takes substantial time before functional testing can be performed. To reduce the amount of ex vivo culture required for functional testing, the use of effective surrogate biomarkers of drug sensitivity would be desirable. While the vast majority of studies on functional testing have used cell viability assay kits that measure metabolic activity—such as the ATP-based, 3-(4,5-dimethylthiazol-2-yl)-2,5-diphenyltetrazolium bromide (MTT) and lactate dehydrogenase (LDH) assay—instead of cell counting, such assays cannot distinguish the activity of cancer cells from other cell types, such as fibroblasts and immune cells. Given the increasing evidence that the tumor microenvironment plays a crucial role, functional testing that can evaluate the efficacy of drugs against individual cancer cells would be desirable. In this review, we summarize the variety of patient-derived ex vivo culture methods available and functional testing with endpoint assays using surrogate biomarkers of drug sensitivity.

## 2. Patient-Derived Ex Vivo Cultures

Characteristics of patient-derived ex vivo culture methods are summarized in Table 1.

### 2.1. Patient-Derived 2D Cultures

**Figure 2:**
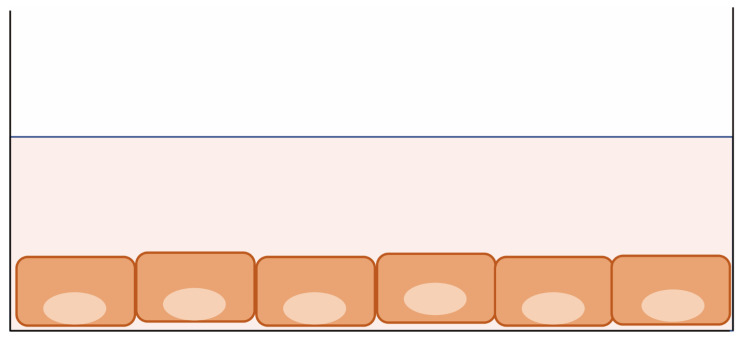
Patient-derived 2D culture. Cancer cells are seeded on a flat plastic surface and cultured with serum-based medium. The cells grow in a monolayer.

Patient-derived 2D cultures (cell lines) are monolayer culture systems on culture plates [11] (Figure 2). Due to their simple and inexpensive handling, cell lines have been indispensable for cancer researchers attempting to understand the mechanisms of cancer progression and drug resistance and for the development of many anti-cancer agents. However, the time-consuming nature of this approach and the low success rate for establishment (<10%) have limited its use as a diagnostic tool for selecting the right drugs for individual patients [30,31]. Furthermore, the expression profiles of cancer cell lines differ from those of cancer cells in vivo and cannot recapitulate the complexity of patients’ cancer biology, which affects their response to anti-cancer drugs [32,33]. Despite such negative implications, many studies have demonstrated a correlation between in vitro drug sensitivity and clinical outcomes using patient-derived 2D cultures in their primary phase [12,13,14,34,35,36,37,38].

### 2.2. Patient-Derived Spheroids

**Figure 3:**
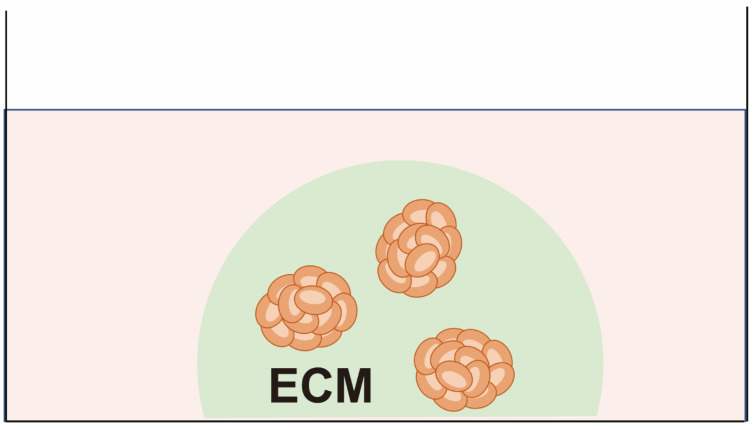
Patient-derived spheroids. Cancer cells are embedded in matrices such as collagen gel or soft agar and cultured with serum-based medium. The cells form 3D aggregates.

To compensate for the drawbacks of cell lines, cancer cells can be cultured in a semi-solid gel or liquid (hanging drop) and floated from a plastic plate to form a 3D structure as spheroids that more closely resemble the tumor microenvironment (Figure 3). Unlike organoids—as described below—spheroids are composed solely of cells with high proliferative activity and lacking the capacity to form tissue type-specific epithelial structures due to the absence of stem cell niche factors in the culture medium [39]. Patient-derived spheroids are used primarily only to test sensitivity to anti-cancer drugs, and they are rarely passaged several times. Spheroid cultures with agar and feeder cells were used for the first attempt at functional testing employing CSRAs [6]. Since then, the feasibility of applying CSRAs clinically has been evaluated by several groups [15,40,41].

Kobayashi and colleagues have developed the CD-DST, in which primary cancer cells are cultured in type 1 collagen under 10% fetal bovine serum (FBS)-containing medium [42]. They were able to demonstrate a correlation between CD-DST results and clinical responses in 11 cases, resulting in a true positive rate of 80%, a true negative rate of 100%, and an accuracy of 91% [16]. Because the feasibility of CD-DST has also been demonstrated by other institutions [10], CD-DST has been covered by health insurance in Japan since 2012.

### 2.3. Patient-Derived Cancer Organoids (PDCOs)

**Figure 4:**
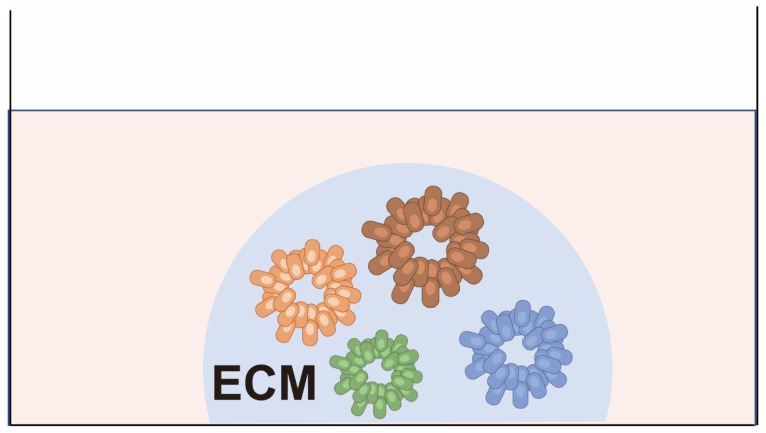
Patient-derived cancer organoids (PDCOs). Cancer cells are embedded in matrices such as matrigel or collagen gel and cultured with medium for tissue-specific adult stem cells. The cells form 3D structures similar to the primary cancer tissue from which they are derived.

In 2009, Sato, Clevers, and colleagues successfully established in vitro cultures of mouse intestinal tract adult stem cells, for which they adopted the term “organoids” because they contained a variety of functionally differentiated cells [43]. Later, in 2011, the same group reported the establishment of organoids from human cancer tissue using a similar approach [17]. Patient-derived cancer organoids (PDCOs) can be established by culturing dissociated patient-derived cancer cells in a semi-solid extracellular matrix (ECM) and expanding them in a medium enriched with stem cell niche factors (Figure 4). PDCOs closely recapitulate the characteristics of the original cancer tissues, such as gene expression profiles and histology. 

A number of attempts have been made to co-culture cancer organoids with various cell types, such as fibroblasts [44,45,46,47], immune cells [48,49,50,51,52,53,54], and other cell types [55,56,57], for the prediction of therapeutic efficacy. PDCOs sometimes contain normal epithelial cells and require weeks to generate a sufficient number of cells for functional testing. Furthermore, the establishment success rates depend on the tissue of origin. Therefore, further improvements and clinical validations are required before PDCOs can be applied clinically.

Despite these limitations, the early results of retrospective functional testing seem to be promising. Since Vlachogiannis et al. demonstrated in 2018 that the drug sensitivity of cancer organoids correlates with patient treatment efficacy [18], a number of groups have conducted retrospective correlation analyses. Although some of those studies found non-significant correlations between ex vivo sensitivity and clinical therapeutic response for certain drugs [58], a number of others found significant correlations [59]. Furthermore, enrichment of cancer cells with stromal cells would be required to test their sensitivity to ICI for functional testing.

Recently, clinical trials have been conducted to verify whether organoid-guided drug selection for functional testing can improve patient outcomes. When the words “cancer” and “organoids” were used to search the ClinicalTrials.gov website, 149 studies were retrieved in August 2023. Among those studies, 60 were interventional clinical studies (Table 2). Whereas Jensen et al. have supported the feasibility of functional testing with PDCOs for the prediction of clinical outcome [19], Ooft et al. have reported that PDCO-guided cancer therapy has only limited clinical benefit and that some challenges still need to be overcome, such as the low success rate of culture establishment and the condition of patients at the last line of standard treatment [20].

PDCOs can be established rapidly and more efficiently than other ex vivo models in expanding culture while maintaining heterogeneity. Therefore, they are highly applicable not only for personalized diagnosis to select optimal drugs based on functional testing but also for research and development of drugs as a biobank reflecting the diversity of cancers. Given that intratumor heterogeneity and cancer evolution in individual patients are the most troublesome aspects of cancer treatment, a “personalized living biobank”, in which multiple cancer organoids are established from a single patient by collecting multiple samples at multiple sites over time, might provide valuable insights into cancer treatment. 

### 2.4. Patient-Derived Explants (PDEs)

**Figure 5:**
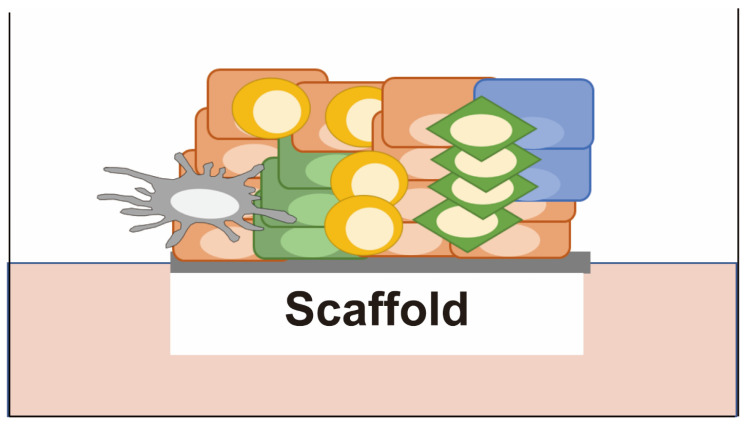
Patient-derived explants (PDEs). Cancer tissues are cultured without disassembly on a scaffold such as a collagen gel or membrane at the air-liquid interface with a serum-based medium. They retain a 3D tissue architecture surrounded by the tumor microenvironment, including fibroblasts and immune cells.

The history of patient-derived explants (PDEs) can be traced back to 1986 when Freeman et al. reported histocultures that had been established from 1.0-mm^3^ pieces of cancer tissue grown on collagen gel soaked in a conventional medium containing 10% FBS [21] (Figure 5). It proved possible to maintain these histocultures for more than 90 days with a success rate of 72% (64/89 tumors), and they retained a 3D tissue architecture surrounded by the tumor microenvironment in vitro and displayed tumorigenicity in nude mice. Correlation analysis of in vitro drug sensitivity and clinical therapeutic efficacy using histocultures was soon referred to as Histoculture Drug Response Assay (HDRA) and involved bulk metabolic measurement as the endpoint using approaches such as the MTT assay, LDH assay, [^3^H]thymidine incorporation, glucose consumption or alamarBlue. The HDRA was subsequently validated for the prediction of therapeutic efficacy in individual patients by a number of studies [22,60,61,62,63,64,65,66,67,68,69,70,71], and it has been covered by health insurance in Japan since 2012. 

Histoculture was further developed to PDEs by Majumder et al. in 2015 [23]. PDEs used matched tumor-stromal matrix proteins (TMP), which were components of the original tissues, and autologous serum in order to recapitulate the tumor environment of individual patients more closely than histoculture. By combining patient clinical data, PDE data, and machine learning, Majumder and colleagues achieved 91.67% specificity and 100% sensitivity for the prediction of responders and non-responders with HNSCC (42 cases) and CRC (13 cases). Karekla et al. Further developed another type of PDE in which tissues were cultured on a membrane with a pore size of 0.4 µm at the air-liquid interface and demonstrated that the PDE response to cisplatin was significantly correlated with patient survival [24]. Rodolfo et al. have also developed original PDEs using a Rotary Cell Culture System (RCCS) in a bioreactor [72].

Because ex vivo cultures of 3D spheroids and PDCOs require disassembly of the patient tumor tissue, it is difficult to capture the full tumor environment in an individual patient. In contrast, PDEs do not require disassembly into single cells and thus recapitulate the original tumor environment, including fibroblasts and immune cells, better than other ex vivo models, thus potentially reproducing the drug response of the tumor. In fact, Straussman et al. Furthermore, Wilson et al. have demonstrated the importance of fibroblasts in drug resistance [73,74]. Furthermore, Voabil et al. have explored the feasibility of patient-derived tumor fragments (PDTF), a form of histoculture, for predicting the efficacy of immune checkpoint inhibitors (ICIs) [75]. On the other hand, PDEs are not suitable for high-throughput drug screening or for biobanking as preclinical models because they have a low capacity for long-term expansion. Furthermore, they exhibit a gradual decline in proliferation rate, which can be a major drawback for functional testing.

### 2.5. Microfluid-Based Culture (Organs on Chips)

**Figure 6:**
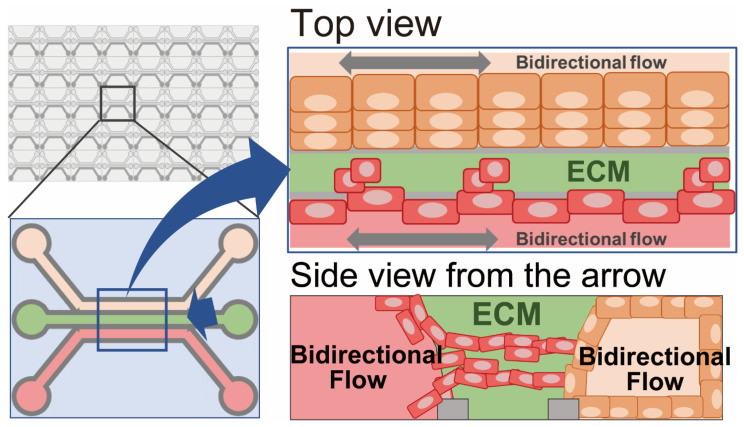
Microfluid-based culture (organs on chips). Cancer cells are co-cultured with multiple cell lineages, such as vascular endothelial cells, fibroblasts, or immune cells, under fluid conditions on a chip platform.

Microfluid-based culture is mainly performed on an “organs on chips” platform, whereby multiple cell lineages are co-cultured under fluid conditions (Figure 6). All of the ex vivo cultures described above are grown in a static medium, whereas fluid-flow cultures are more physiological in that they mimic the constant flow present in living tissues, where nutrients, oxygen, and metabolites are circulating, and dead cells are being removed. Fluidic shear stress in organ chips, also generated using moderate medium flow, promotes the maturation of the cells on the chip. Furthermore, microfluid-based cultures recapitulate the in vivo situation more closely by incorporating not only a single-cell lineage but also microenvironment-related cells and a suitable ECM.

In 2010, Huh et al. first reported a microfluid-based culture for the physiological alveolus-capillary unit of the lung in an organ-on-a-chip platform [25]. In their organ-on-a-chip, there are two cell compartments, one for alveolar epithelial cells and the other for lung microvascular endothelial cells. The two compartments are separated by an ECM-coated microporous permeable membrane and maintain a tissue-specific environment for each cell lineage, i.e., air on the alveolar side and fluidic flow on the vascular side. To simulate the effect of breathing, the lung tissue chip is further subjected to cyclic vacuum application that induces cyclic stretching of the cell-attached membrane. 

Such organs-on-chips have developed into various types of in vitro tissue constructions for both physiological [76] and cancerous tissues [77,78,79,80,81,82,83]. Gheibi et al. were the first to report the application of PDX-derived and primary bladder cancer cells for a microfluidic device [77]. Using this device, they demonstrated that the cancer cells continued to grow for 30 days to become millimeter-size spheroids. Several recent studies have adapted such organs-on-chips for drug screening using patient-derived cancer cells [26,84,85,86]. Analysis of the correlation between drug sensitivity in ex vivo culture and clinical response would be warranted.

Unlike other ex vivo cultures, patient-derived cancer cells for microfluidic cultures need to be embedded in the device and are mostly co-cultured with other cell types. As a consequence, bulk-metabolic assays are not suitable for determining drug efficacy in microfluidic cultures, and imaging analysis has developed as an endpoint assay. Particularly, organ-on-chip-based functional testing for immune-checkpoint inhibitors involving co-culture with immune cells [27] is being developed.

### 2.6. Micro-Organospheres (MOSs)

**Figure 7:**
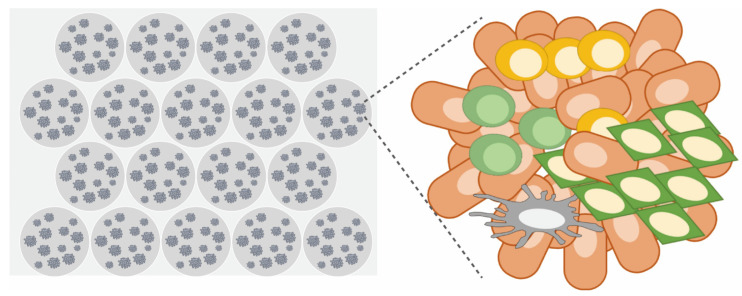
Micro-organospheres (MOSs). An advanced version of patient-derived cancer organoids. Cancer cells are digested and 3D-cultured in a microfluidic-based Matrigel droplet using a benchtop machine. At the primary stage, they contain immune cells, making it possible to predict the efficacy of ICI.

Micro-organospheres (MOSs) are a form of ex vivo culture in which an automated microfluidic device is combined with primary organoid culture. MOSs are an automatic microfluidic droplet platform by which very small samples of cancer tissue, such as endoscopic and needle-core biopsies, can be adapted for high-throughput functional testing in a short period of time (within 10–14 days, making them valid for clinical treatment decision-making) (Figure 7). As described in detail by Ding et al. Furthermore, Wang et al. [28,29], cancer cells are suspended in matrigel and mixed with oil to form MOS droplets. After removal of the oil using demulsification—also a novel approach developed by the group—MOS droplets containing 20–100 organoids are cultured until they reach a size of 250–450 µm and then subjected to functional testing. Ding et al. achieved functional testing with 119 FDA-approved drugs using as few as 15,000 patient-derived cells within 13 days (9.9 days on average) of obtaining 18-gauge core biopsies from 8 patients with metastatic colorectal cancers. They successfully established MOSs from all eight patients (100%) and demonstrated that survival outcomes tended to be correlated with MOS-based functional testing. A point of further interest was that MOSs at passage 0 retained some stromal cells of the original tumor tissue with functional immune cells, thus enabling functional testing of immune checkpoint inhibitors.

## 3. Endpoint Assays for Functional Testing

Characteristics of endpoint assays are summarized in Table 3.

### 3.1. ATP-Based Bulk Assay

The most common endpoint drug sensitivity assay for ex vivo cultures is the measurement of intracellular ATP, which is a marker for the presence of metabolically active cells. Various kits for this are commercially available, such as the CellTiter-Glo^®^ 3D Cell Viability Assay (Promega, Madison, WI, USA). ATP-based cell viability assay uses intracellular ATP as a surrogate bioindicator of the number of viable cells in a culture, having been first demonstrated by Crouch et al. in 1993 [109]. Over the last 5 years, many studies using this endpoint assay have demonstrated a significant correlation between ex vivo drug sensitivity and clinical therapeutic effects [18,58,59,110]. The ATP-based assay is more sensitive than other metabolic activity-related assays conventionally used for 2D cell cultures, such as the alamarBlue, MTT, LDH, and SDH assays, and can detect as few as 15 viable cells in one well of a 384-well plate [111]. Furthermore, this endpoint assay has been adapted to automated and high-throughput drug sensitivity assays by a number of research groups. However, as this assay usually measures bulk ATP in one well at a single time point, it may underestimate any intra-tumor variability of growth speed or drug response when other cell types are present in co-culture.

### 3.2. Dynamic BH3 Profiling (DBP)

The adaption of dynamic BH3 profiling (DBP) to functional testing was first reported by Montero et al. in 2015 [88]. DBP evaluates susceptibility to drugs by measuring the dynamics of mitochondrial polarization—an early event of apoptosis—upon treatment with anti-cancer drugs. To detect mitochondrial polarization, JC-1 staining, which indicates the integrity of the mitochondrial membrane or efflux of cytochrome *c*, was used as a surrogate marker. The roughly 20-amino-acid BH3 domain of BIM, a pro-apoptotic protein, has the ability to induce mitochondrial depolarization, thus lowering the apoptotic threshold. In this assay, to optimize the dynamics of changes in mitochondrial polarization caused by anti-cancer drug treatment, the cells are treated with an optimized concentration of the BH3 domain peptide, at which depolarization barely occurs. The additive effect of a test drug on BH3 domain peptide-dependent depolarization is evaluated as the drug sensitivity. In other words, if the BH3 domain peptide-dependent depolarization is increased using pretreatment with a certain drug, susceptibility to the drug will be high; conversely, if the depolarization is less affected by the pretreatment, the drug susceptibility will be low. Usually, treatment with anti-cancer drugs alone has only a limited effect on cytochrome c release and JC-1-positive cells. The BH3 domain peptide reduces mitochondrial polarization, thus making it easier to detect any changes induced using the drug.

Because DBP detects very early changes in the apoptotic response, it can be performed rapidly (typically < 24 h), allowing its application to early ex vivo cultures from patients. Although specialized materials, equipment, and techniques are required, this method has been validated by other groups [89,90] as well as the original inventor [91]. Since induction of apoptosis is especially important for the efficacy of anti-cancer drugs in hematological cancers, DBP is particularly useful for this type of cancer. Although the usefulness of DBP for solid tumors is doubtful because non-apoptotic cell death is also a frequent effect of anti-cancer drugs [112], a recent study has also adapted DBP for the prediction of therapeutic efficacy against solid tumors [91]. Furthermore, Potter et al. investigated the potential use of DBP for the prediction of BH3 mimetics [113]. However, this requires further validation.

### 3.3. Optical Metabolic Imaging (OMI) and Fluorescence Lifetime Imaging Microscopy (FLIM) Based on Metabolite Autofluorescence

The metabolic activity of cancer cells is known to be an early predictor of cellular behavior in response to treatments with anti-cancer drugs. Optical metabolic imaging (OMI) and fluorescence lifetime imaging microscopy (FLIM), which are both endpoint assays, use the cellular autofluorescence intensities of metabolites such as NAD(P)H and FAD for measuring metabolic activity in individual cells after drug treatment and can predict the therapeutic response of patients. The intercellular metabolic cofactor NAD(P)H (the reduced form of nicotinamide adenine dinucleotide) is present in both protein-bound and protein-free forms in cells, and its status affects the rate of decline of its autofluorescence. Protein-bound NAD(P)H typically exhibits a longer lifetime than free NAD(P)H.

For OMI, the redox ratio is calculated by dividing the NAD(P)H intensity by the FAD intensity [92]. For FLIM, as reported by Morelli et al., the NAD(P)H lifetime is measured to demonstrate metabolic activity in living cells and tissues [93]. Trinh et al. have demonstrated that the NAD(P)H fluorescence lifetime (NAD(P)H bound/free ratio) increases as cancer cells become less proliferative as a result of drug treatment [114], thus indicating their drug sensitivity. Similarly, Yan et al. have reported a FLIM based on lipofuscin-like autofluorescence, which shows acute accumulation during the cell death process and can distinguish necrosis from apoptosis [94].

Although these assays require special equipment and complicated mathematical modeling to detect and evaluate metabolite autofluorescence, they have certain advantages that may outweigh such disadvantages. For example, these endpoint assays can be performed rapidly after drug treatment since the resulting metabolic shift precedes any change in cell viability. Furthermore, since the autofluorescence of metabolites is detectable at the single-cell level in 3D culture without the use of dyes or disruption of ex vivo cultures, these assays can be performed over time to assess spatial heterogeneity in culture.

### 3.4. Fluorescent Dyes (Calcein-AM, Hoechst and Propidium Iodide)

Several groups have proposed the use of conventional fluorescent dyes, such as calcein-AM, Hoechst, and propidium iodide (PI), to evaluate cell viability for the prediction of chemotherapeutic response. Li et al. demonstrated that calcein-AM was able to detect the viability of 3D culture more clearly than Hoechst and 5-(and-6)-carboxyfluorescein diacetate, succinimidyl ester (CFDA SE) and optimized the concentration of calcein-AM to 2 µM for a 60 min assay [95]. Bode et al. have developed a fast, simple, and quantitative method to detect cell death over time by adding Hoechst and PI to the culture medium without disrupting cancer cells and used the ratio of the PI/Hoechst signals to calculate cell death in organoids [96]. While the endpoint assays using these dyes are straightforward for the evaluation of cell viability over time without any need for specific equipment, techniques, or lysis of ex vivo cultures, any correlation between the results obtained from patient-derived ex vivo cultures and actual clinical outcomes remains to be confirmed.

### 3.5. Mass Accumulation Rate (MAR) Assay Using a Suspended Microchannel Resonator (SMR)

A clear scheme of this technique has been presented by Stockslager et al. [97]. Mass accumulation rate (MAR) can be determined by measuring the buoyant mass of single cells over time (every 15 s for approximately 15 min for each single cell) on a suspended microchannel resonator (SMR), which is a cantilever-based microfluidic mass sensor that measures the buoyant mass of live single cells with a resolution close to 50 fg under the conditions of culture. Stevens et al. have demonstrated that the change in MAR induced by a drug represents chemotherapeutic efficacy in both an in vitro patient-derived glioblastoma (GB) cell line and an in vivo mouse ALL model [98].

The original flow device of the SMR had a single-lane format and limited throughput for drug testing because at least 1–2 h was typically required for measuring the effects of each drug on a sufficient number of single cells. To achieve higher throughput, Stockslager et al. developed a microfluidic device containing 16 SMRs connected fluidically in parallel (SMR array) and operated simultaneously on the same microfluidic chip [99]. Using the SMR array in combination with patient-derived neurosphere models, Stockslager et al. demonstrated that MAR was able to detect subtle changes in the mass of individual drug-treated cancer cells as a surrogate biomarker of the anticipated patient treatment response [97]. In a retrospective study, they also demonstrated by MAR assay that the efficacy of temozolomide on ex vivo cultures derived from GB patients was correlated more closely with matched patient survival than that estimated from the ATP-based bulk assay [97].

Although the MAR assay needs specialized equipment and single-cell generation before the assay, it does have certain advantages. For example, it does not need long-term expansion of ex vivo cultures, thus allowing rapid prediction of chemotherapeutic efficacy. Additionally, since cells remain viable after the MAR assay, they can be used for downstream molecular and functional assays. Furthermore, since the MAR assay evaluates drug sensitivity at the single-cell level, any heterogeneity of sensitivity or growth speed can be characterized for individual cells in each culture.

### 3.6. Live Cell Imaging-Based Endpoint Assay

To evaluate the drug sensitivities of 3D organoid cultures, Deben, Le Compte, and colleagues have developed organoid bright field identification-based therapy screening (OrBITS) involving a combination of widefield organoid images and automated live-cell image analysis software [100,101]. While this assay requires the expansion of organoids from each patient, any heterogeneity of drug sensitivity can be quantified over time by single-organoid analysis to identify potentially resistant clones. Such endpoint assays with widefield live cell imaging and high-content imaging are high-throughput compatible and have also been adapted by several groups [102,115,116]. Correlations between the results of these endpoint assays and clinical outcomes remain to be analyzed.

### 3.7. Immunostaining-Based Endpoint Assays

As it becomes more widely accepted that the tumor microenvironment influences sensitivity to anti-cancer drugs, functional testing using co-cultures or primary cultures containing other cell types, such as cancer-associated fibroblasts (CAFs) and immune cells, is being developed. With such ex vivo cultures, several groups have proposed immunostaining-based drug sensitivity tests as endpoint assays because endpoint assays that evaluate bulk metabolic activity, such as ATP-based, MTT, and LDH assays, cannot distinguish the activity of cancer cells from other cell types. As well as their ability to distinguish cancer cells from non-cancer cells, immunostaining-based endpoint assays have the advantage of requiring fewer cells, allowing diagnosis of individual cells. This is particularly important when analyzing solid tumors since only very small samples of cancer tissue are available from inoperable patients. 

To determine the drug sensitivity of ex vivo cultures derived from lung cancer biopsies, Kodack et al. stained PDEs with CK8/CK18 (a marker of epithelial cells) to quantify viable cancer cells in PDEs after drug treatment [103]. For the quantification of positive signals, they used a high-content imager (ImageXpress Micro XL, Molecular Devices, San Jose, CA, USA) and its compatible software (MetaXpress software, Molecular Devices). They first used a homogeneous cancer cell line to confirm that immunostaining-based drug sensitivity testing could be performed with as few as 100 cells and that the drug-response curve of the assay was comparable to that of the ATP-based bulk assay. They subsequently established lung cancer ex vivo cultures using an original method involving feeder cells and showed that the primary cultures contained a mixture of cell types other than cancer cells. Using these ex vivo cultures, they demonstrated a correlation between the results of the immunofluorescence-based functional assays and the patient’s response to chemotherapy, although the number of patients analyzed was limited.

In histoculture and PDE, where patient tissues are sectioned and directly cultured, endpoint assays of metabolic activity in bulk cultures, such as SDH, MTT, and LDH, used to be the mainstay. However, as PDEs include fibroblasts, immune cells, and vascular cells that constitute the microenvironment, it was difficult to distinguish the sensitivity of cancer cells from that of other cells based on bulk-metabolic activity. Pritchard and colleagues have been developing an immunostaining-based method for spatial profiling of PDE drug sensitivity [24,87,104]. They first demonstrated that immunohistochemistry (IHC) for cleaved PARP (cPARP), a marker of apoptosis, in PDE after treatment with cisplatin was correlated with patient survival [24]. Then, to separate the signals of cancer cells from those of other cells, they developed multispectral imaging involving chromogenic IHC for Ki67, cPARP, and pan-cytokeratin (CK) using serial sections of PDEs, which were then subjected to in silico image alignment analysis [87]. They further advanced their spatial profiling and developed a multiplex immunofluorescence approach to compare the PDE drug response with actual clinical outcomes [104]. They also demonstrated the potential use of this endpoint assay to assess the efficacy of pembrolizumab, an ICI.

Rodolfo et al. used a bioreactor to establish PDEs with prolonged survival and demonstrated their immunologic response to PD-1 treatment by immunostaining, suggesting the potential of PDEs for prediction of ICI response [72]. Ding et al. have also developed an immunostaining-based endpoint assay to detect the T-cell immune response against tumor cells by using MOSs containing both patient-derived tumor organoids and their associated immune cells [28]. Thus, endpoint assays based on immunostaining have the potential to take the tumor microenvironment into account, although special equipment for quantification, such as high-content imaging and a multiplex immunofluorescence approach, are necessary to achieve this assay. Further validation studies to compare the drug sensitivity of ex vivo cultures and clinical response will be required. Although only limited studies have used organs on chips to investigate the association of ex vivo drug response with clinical outcome, immunostaining-based endpoint assays would be a practical option since these ex vivo cultures mostly involve co-culture.

### 3.8. Immunofluorescence Detection of Drug-Specific Response Biomarkers

In association with immunostaining-based endpoint assays, some groups have utilized sensitivity-dependent intracellular changes, such as accumulated proteins or phosphorylation status, after treatment with radiation or drugs as surrogate biomarkers of sensitivity. Immunofluorescence-based detection of such biomarkers also has the advantage of requiring fewer cells. Hill et al. have developed a functional assay using PDCOs of ovarian cancer to predict the efficacies of PARP, CHK1, and ATR inhibitors and carboplatin [105]. It is known that genomic alterations affecting genes associated with the DNA damage response (DDR) increase the degree of sensitivity to inhibitors that target DNA repair defects [117,118,119,120]. However, the functional consequences of individual genomic alterations in DNA-repair genes are complicated and remain incompletely characterized. Using PDCOs, Hill and colleagues demonstrated that the immunohistochemical status of RAD51 foci, a hallmark of homologous recombination (HR) functionality, after short-term irradiation was able to predict sensitivity to a PARP inhibitor more precisely than genomic alterations in HR-related genes. Furthermore, they demonstrated that functional testing for replication fork instability in PDCOs was able to predict sensitivity to CHK1 and ATR inhibitors and carboplatin. Importantly, their data suggested that functional testing using PDCOs was able to predict sensitivity to particular drugs more precisely than diagnosis based on genomic alterations. These assays can be performed at the single-cell level, thus allowing testing with small amounts of organoids. Compadre et al. have also adapted immunostaining detection of RAD51 foci induced by irradiation in patient-derived ex vivo cultures to the patients’ response to chemotherapy with platinum agents [106].

Similarly, our group has proposed a method for predicting the effect of cytotoxic drugs using c-Jun activation after cisplatin treatment as a surrogate biomarker [107] based on the fact that stress-responsive MAPK (JNK) is highly activated in cancer cells that are more sensitive to DNA-damaging cytotoxic drugs. We showed that c-Jun activation in PDCOs after cisplatin treatment tended to be correlated with the effect of neoadjuvant chemotherapy (docetaxel, cisplatin, and 5-fluorouracil) in matched patients. Additionally, we have reported that a reduced phosphorylation level of ribosomal S6 (pS6) after treatment with MEK inhibitor is a potential surrogate biomarker of MEK inhibitor efficacy in cell lines as well as PDCOs [108,121]. Of note, Corcoran et al. had previously reported the potential application of pS6 reduction as a biomarker for the efficacy of MEK and RAF inhibition in melanoma [122]. Despite the need to identify a suitable biomarker for each drug, these surrogate biomarkers are detectable by immunostaining at the single-cell level and could be used as a simple and robust immunostaining-based endpoint assay.

## 4. Conclusions and Future Directions

In solid tumors, surgically resected tumor tissue is the most abundant source of cancer cells. However, patients with recurrent or metastatic tumors usually do not undergo surgery, and chemotherapy may be the only option for those with inoperable tumors. In addition, patients who are scheduled for neoadjuvant chemotherapy receive chemotherapy prior to undergoing surgery. Therefore, for patients with inoperable cancer or those who receive preoperative chemotherapy, cancer cells can only be obtained from a small amount of diagnostic material, such as endoscopic, fine-needle aspirates (FNA), needle cores, and liquid biopsies. To provide a rapid diagnosis from such small materials for individual patients, rapid methods for the preparation of ex vivo cultures and endpoint assays are essential.

It is likely that many of the studies for functional testing aim to accomplish high-throughput screening to select an optimal candidate drug from hundreds of possible candidates for any individual patient. However, without functional testing, it is difficult to choose the best one, even if several approved drugs are available. Furthermore, NGS-based diagnosis is not suitable for predicting the effects of cytotoxic drugs despite their predominant use as standard therapy for many tumor types. In such a clinical context, low-throughput drug screening focused on only a few approved drugs would arguably be more practical and beneficial than complicated high-throughput screening.

The usefulness of functional testing may differ depending on the type of anti-cancer drug [58], i.e., the idea that a specific combination of ex vivo culture and endpoint assay might predict the efficacy of all types of anti-cancer drugs may be incorrect. For example, DBP predicts drug efficacy by evaluating the early apoptotic response after drug treatment. However, not all anti-cancer drugs exert their effects in an apoptosis-dependent manner, especially in solid tumors [112].

Many reviews on patient-derived ex vivo cultures have discussed their application to both diagnostic materials and biobanks. If the aim is to apply ex vivo culture to diagnostic materials for prediction of drug efficacy in individual patients, it might be better to use PDEs or other primary ex vivo cultures without passaging, such as MOSs, because they include not only cancer cells but also cells in the tumor microenvironment and are capable of predicting the efficacy of ICIs. Given increasing evidence that immunotherapy has the potential to become a main treatment for cancer, functional testing using models with a functional immune microenvironment is extremely important. Ben-David and colleagues have shown that repeated passaging changes the properties of patient-derived cancer cells, suggesting the need to consider genomic evolution and its associated functional consequences in any type of living cancer model [123,124,125]. In contrast, if the aim is to apply ex vivo cultures as a living biobank [126] for research and development, passaging and expansion are needed. For this purpose, PDCOs would appear to be the best approach.

As is true for any diagnostic method, it is difficult to overcome variations in diagnostic outcomes due to intra-tumor heterogeneity. While it is difficult for the ATP-based assay to reflect the heterogeneity of individual cell chemosensitivity, immunostaining-based endpoint assays can detect such intra-tumoral heterogeneity. In the future, it may become possible for functional testing to quantify such heterogeneity.

For the clinical application of functional testing, it is crucial to design any clinical study by consultation with a medical oncology team and set stringent criteria for patient enrollment and endpoint assays, as up to now, most of the criticism of ex vivo-guided clinical trials has focused on inappropriate organization of the study design [7,8,9]. In addition, simplification of the handling and devices would make such functional testing easier to apply clinically.

## Figures and Tables

**Table 1:** Ex vivo cultures for functional testing.

Ex Vivo Cultures	Heterogeneity ^a^	Co-Culture ^b^	Medium And ECM Gels	Success Rate for Primary Culture ^c^	Expansion for Biobank ^d^	Key Articles ^e^
**Patient-derived 2D cultures**	Low	Exogeneous components	Serum-based	High, but depending on the tissue type	Yes	Scherer et al., 1953 [11] Cree et al., 1996 [12] Andreotti et al., 1995 [13] Hunter et al., 1993 [14]
**Patient-derived spheroids**	Low	Exogeneous components	Serum-based medium with ECM gel, such as collagen and soft agar	High, but depending on the tissue type	No	Hamburger et al., 1977 [6] Von Hoff et al., 1983 [15] Kobayashi et al., 1997 [16]
**Patient-derived cancer organoids (PDCOs)**	High	Exogeneous and patient-derived components	Stem cell culture-based medium with ECM gel, such as Matrigel and collagen	High, but depending on the tissue type	Yes	Sato et al., 2011 [17] Vlachogiannis et al., 2018 [18] Jensen et al., 2023 [19] Ooft et al., 2021 [20]
**Patient-derived explants (PDEs),** **Histoculture**	Very high	Patient-derived components	Serum-based medium with scaffold, such as sponge and transwell	High, but depending on the tissue type	No	Freeman et al., 1986 [21] Furukawa et al., 1995 [22] Majumder et al., 2015 [23] Karekla et al., 2017 [24]
**Microfluid-based culture (Organs on chips)**	-	Exogeneous components	Microfluidic medium with a few compartments for co-culture	High, but depending on the tissue type	No	Huh et al., 2010 [25] Schuster et al., 2020 [26] Jenkins et al., 2018 [27]
**Micro-organospheres (MOSs)**	High	Patient-derived components	Stem cell culture-based medium with Matrigel	High, but depending on the tissue type	Yes	Ding et al., 2022 [28] Wang., 2022 [29]

^a^; Intra-tumor heterogeneity. ^b^; Co-culture with other cell types. ^c^; Focus only on primary culture success rate for functional testing. ^d^; Biobank for drug R&D. ^e^; Selected based on early reports and clinically related studies.

**Table 2:** Currently or recently registered clinical trials on organoid-guided treatment.

Study Title	NCT Number	Phases	Enrollment	Start Date	Study Status
PTCs-based Precision Treatment Strategy on Recurrent High-grade Gliomas	NCT05473923	EARLY_PHASE1	30	2022/8/12	RECRUITING
[18F]Fluoroestradiol-PET/CT Companion Imaging Study to the FORESEE Trial	NCT04727632	EARLY_PHASE1	6	2021/3/31	RECRUITING
Pilot Trial for Treatment of Recurrent Glioblastoma	NCT05432518	EARLY_PHASE1	10	2023/6/27	RECRUITING
Modulation of Ciliogenesis in Glioma Stem Cells	NCT05772767	NA	80	2021/2/15	RECRUITING
Patient-derived Organoids Drug Screen in Pancreatic Cancer	NCT05351983	NA	50	2022/9/22	RECRUITING
Evaluation and Comparison of the Growth Rate of Pancreatic Cancer Patient-derived Organoids	NCT03990675	NA	50	2018/12/1	UNKNOWN
CPCT-05 Biopsy Protocol Patient Selection	NCT01904916	NA	195	2014/1	TERMINATED
Systemic Neoadjuvant and Adjuvant Control by Precision Medicine in Rectal Cancer	NCT04842006	NA	93	2021/12/20	RECRUITING
Selecting Chemotherapy With High-throughput Drug Screen Assay Using Patient Derived Organoids in Patients With Refractory Solid Tumours (SCORE)	NCT04279509	NA	35	2019/5/29	UNKNOWN
Individualized Precision Treatment Based on Ovarian Cancer Organoid Model	NCT05813509	NA	30	2022/12/1	RECRUITING
OPPOSITE: Outcome Prediction Of Systemic Treatment in Esophagogastric Carcinoma	NCT03429816	NA	40	2018/4/15	ACTIVE_NOT_ RECRUITING
Organoids From Metastases of Prostate Cancer	NCT03952793	NA	1	2019/12/4	TERMINATED
The Safety and Feasibility of Costal Bone Marrow Aspiration During Thoracic Surgery	NCT05251805	NA	10	2023/3/20	RECRUITING
Establishing Organoids From Metastatic Pancreatic Cancer Patients, the OPT-I Study.	NCT03500068	NA	30	2017/9/4	UNKNOWN
Establishment of Pancreas Cancer and Cancer-associated Fibroblast Using EUS-guided Biopsy Samples	NCT05571956	NA	50	2020/7/1	RECRUITING
Evaluation of ex Vivo Drug Combination Optimization Platform in Recurrent High Grade Astrocytic Glioma	NCT05532397	NA	10	2023/2/17	RECRUITING
Clinical Study on Drug Sensitivity Verification or Prediction of Therapy for Breast Cancer by Patient-Derived Organoid Model	NCT03544047	NA	50	2019/1/1	UNKNOWN
In Vitro Organoid Drug Sensitivity-Guided Treatment for Metastatic Pancreatic and Gastric Cancer	NCT05842187	NA	20	2023/3/3	RECRUITING
Primary Organoid Models and Combined Nucleic Acids Therapeutics for Anti-HPV Treatments	NCT04278326	NA	50	2020/3/6	RECRUITING
Functional Precision Oncology for Metastatic Breast Cancer	NCT04450706	NA	15	2021/2/16	RECRUITING
Record Voxel Rate Nonlinear Optical Microscope to Unravel Brain Connectome and Signaling-Establish Reliably Electrophysiological Readouts From Human-induced Pluripotent Stem Cells (hiPSCs)-Derived Cerebral Organoids and Surgically Dissected Human Live Brains	NCT05921786	NA	500	2023/5/1	RECRUITING
UZ/KU Leuven Program for Post-mortem Tissue Donation to Enhance Research	NCT04531696	NA	100	2020/11/30	RECRUITING
Stereotactic Body Radiation Therapy for Unresectable Perihilar Cholangiocarcinoma	NCT03307538	NA	6	2017/11/6	COMPLETED
The Clinical Efficacy of Drug Sensitive Neoadjuvant Chemotherapy Based on Organoid Versus Traditional Neoadjuvant Chemotherapy in Advanced Rectal Cancer	NCT05352165	NA	192	2023/1/1	NOT_YET_ RECRUITING
A Platform of Patient Derived Xenografts (PDX) and 2D/3D Cell Cultures of Soft Tissue Sarcomas (STS)	NCT02910895	NA	40	2017/9/9	RECRUITING
Markers to Evaluate the Efficacy of PH-based Regimen as a Neoadjuvant Therapy for Operable HER2 Positive Breast Cancer	NCT04281641	NA	94	2020/4/21	RECRUITING
Patient-Derived Organoids for Rectal Cancer	NCT04371198	NA	20	2020/9/18	COMPLETED
Precision Chemotherapy Based on Organoid Drug Sensitivity for Colorectal Cancer	NCT05832398	NA	186	2023/5/1	RECRUITING
Grafts of GSCs Into Brain Organoids for Testing Anti-invasion Drugs	NCT05772741	NA	160	2018/12/3	RECRUITING
Prospective Multicenter Study Evaluating Feasibility and Efficacy of Tumor Organoid-based Precision Medicine in Patients With Advanced Refractory Cancers	NCT05267912	NA	1919	2022/1/19	RECRUITING
Engineering Immune Organoids to Study Pediatric Cancer	NCT05890781	NA	44	2023/5/12	RECRUITING
TCR-T Cell Immunotherapy of Lung Cancer and Other Solid Tumors	NCT03778814	PHASE1	30	2018/12/1	RECRUITING
Testing ONC201 to Prevent Colorectal Cancer	NCT05630794	PHASE1	24	2023/5/13	NOT_YET_ RECRUITING
Quadratic Phenotypic Optimisation Platform (QPOP) Utilisation to Enhance Selection of Patient Therapy Through Patient Derived Organoids in Breast Cancer	NCT05177432	PHASE1	26	2021/12/6	RECRUITING
Birinapant and Carboplatin in Treating Patients With Recurrent High Grade Ovarian, Fallopian Tube, or Primary Peritoneal Cancer	NCT02756130	PHASE1|PHASE2	0	2018/8/1	WITHDRAWN
Optimizing and Personalising Azacitidine Combination Therapy for Treating Solid Tumours QPOP and CURATE.AI	NCT05381038	PHASE1|PHASE2	10	2022/6	NOT_YET_ RECRUITING
Cancer Preventive Vaccine Nous-209 for Lynch Syndrome Patients	NCT05078866	PHASE1|PHASE2	45	2022/11/10	RECRUITING
Cisplatinum and Everolimus in Patients With Metastatic or Unresectable NEC of Extrapulmonary Origin	NCT02695459	PHASE2	39	2016/3/30	UNKNOWN
Pancreatic Adenocarcinoma Signature Stratification for Treatment	NCT04469556	PHASE2	150	2020/10/14	RECRUITING
Treatment of Newly Diagnosed Patient’s With Wilm’s Tumor Requiring Abdominal Radiation Delivered With Proton Beam Irradiation	NCT04968990	PHASE2	95	2021/8/19	RECRUITING
Trifluridine/Tipiracil and Irinotecan for the Treatment of Advanced Refractory Biliary Tract Cancer	NCT04072445	PHASE2	28	2019/10/18	ACTIVE_NOT_ RECRUITING
Functional Precision Oncology to Predict, Prevent, and Treat Early Metastatic Recurrence of TNBC	NCT05464082	PHASE2	80	2023/1/6	RECRUITING
A Trial With Chemotherapy, Immunotherapy, and Radiotherapy for Patients With Newly Diagnosed Stage IV Small Cell Lung Cancer	NCT04951115	PHASE2	42	2021/7/12	RECRUITING
Niraparib Maintenance Treatment in mCRC With a Partial Complete Response After Oxaliplatin-based Induction Therapy	NCT05412706	PHASE2	46	2023/6/1	NOT_YET_ RECRUITING
Oral Iloprost for the Prevention of Lung Cancer In Former Smokers	NCT05411107	PHASE2	80	2023/12/1	NOT_YET_ RECRUITING
Patient-derived-organoid (PDO) Guided Versus Conventional Therapy for Advanced Inoperable Abdominal Tumors	NCT05378048	PHASE2	0	2022/7/4	WITHDRAWN
Atezolizumab + Cabozantinib in Patients w/ Metastatic, Refractory Pancreatic Cancer	NCT04820179	PHASE2	29	2021/10/12	RECRUITING
Organoids Predict Therapeutic Response in Patients With Multi-line Drug-resistant Non-small Cell Lung Cancer	NCT05669586	PHASE2	50	2023/2/1	RECRUITING
The Study of Gemcitabine Plus Nab-Paclitaxel in Combination With Pegvorhyaluronidase Alfa (PVHA; PEGPH20) and Pembrolizumab as Front-line Treatment for Metastatic Pancreatic Adenocarcinoma.	NCT04045730	PHASE2	0	2019/11/15	WITHDRAWN
High Dose Vitamin C Intravenous Infusion in Patients With Resectable or Metastatic Solid Tumor Malignancies	NCT03146962	PHASE2	61	2017/3/29	COMPLETED
Liposomal iRInotecan, Carboplatin or oXaliplatin for Esophagogastric Cancer	NCT03764553	PHASE2	310	2019/5/1	RECRUITING
Imatinib as Pre-operative Anti-Colon Cancer Targeted Therapy	NCT02685046	PHASE2	5	2016/4	TERMINATED
PaTcH Study: A Phase 2 Study of Trametinib and Hydroxychloroquine in Patients With Metastatic Refractory Pancreatic Cancer	NCT05518110	PHASE2	22	2023/5/31	RECRUITING
Early-Line Anti-EGFR Therapy to Facilitate Retreatment for Select Patients With mCRC	NCT04587128	PHASE2	110	2020/10/19	RECRUITING
Guiding Instillation in Non Muscle-invasive Bladder Cancer Based on Drug Screens in Patient Derived Organoids	NCT05024734	PHASE2	33	2022/11/17	RECRUITING
Study to Investigate Outcome of Individualized Treatment in Patients With Metastatic Colorectal Cancer	NCT05725200	PHASE2	40	2022/9/27	RECRUITING
Organoid-Guided Adjuvant Chemotherapy for Pancreatic Cancer	NCT04931394	PHASE3	200	2021/6/1	RECRUITING
Precise Therapy for Refractory HER2 Positive Advanced Breast Cancer	NCT05429684	PHASE3	120	2021/1/1	RECRUITING
Individualized Locoregional Treatment of Initially Biopsy-proven Node-positive Breast Cancer After Primary Systemic Therapy	NCT04281355	PHASE3	0	2021/1/1	WITHDRAWN
Organoid-Guided Chemotherapy for Advanced Pancreatic Cancer	NCT04931381	PHASE3	100	2021/6/1	RECRUITING

“Cancer” and “Organoids” were used to search the ClinicalTrials.gov website (July 2023). All of the trials are interventional.

**Table 3:** Endpoint assays for functional testing.

Endpoint Assays	Surrogate Biomarkers for Drug Efficacy	Bulk or Individual Cells ^a^	Serially Detectable ^b^	Detection Method	Use Specific Equipment	Unique Points	Key Papers
**ATP-based bulk assay**	ATP	Bulk	No	Luminometer	No	Sensitive, most widely used among the endpoint assays	Ooft et al., 2019 [58] Yao et al., 2020 [80] van de Wetering et al., 2015 [87]
**Dynamic BH3 profiling (DBP)**	Mitochondrial depolarization ^c^	Bulk	No	Fluorescent detector	Yes (e.g., MACSQuant and MetaXpress)	Detect early apoptotic event	Montero et al., 2015 [88] Schroeder et al., 2021 [89] Manzano-Munoz et al., 2022 [90] Bhola et al., 2020 [91]
**Optical metabolic imaging (OMI)** **and fluorescence lifetime imaging microscopy (FLIM)**	Metabolites’ autofluorescence, such as NAD(P)H, FAD and lipofuscin	Individual cells	Yes	Fluorescent detector	Yes (e.g, titanium:sapphire laser, custamized filter cube, SPC-150 [Becker & Hickl], SPCImage)	Detectable over time at single cell level	Pasch et al., 2019 [92] Morelli et al., 2022 [93] Yan et al., 2022 [94]
**Fluorescent dye-based assay**	Fluorescence of Calcein-AM, Hoechst and PI	Individual cells	Yes	Fluorescent detector	No	Cost effective	Li et al., 2022 [95] Bode et al., 2019 [96]
**Mass accumulation rate** **(MAR) assay by suspended** **microchannel resonator (SMR)**	Buoyant mass	Individual cells	No (but non-invasive)	Single cell mass measurement by resonance frequency signal	Yes (homemade SMR)	Expansion of culture is not required.	Stevens et al., 2016 [97] Stockslager et al., 2019 [98] Stockslager et al, 2021 [99]
**Live cell imaging based assay**	Live cell images	Individual cells	Yes	Microscope	Yes (e.g., BioTek Cytation 5 Cell Imaging Multimode Reader, CellProfiler, ImageXpress Micro XLS system)	Adapted for PDCOs	Deben et al., 2023 [100] Le Compte et al., 2022 [101] Herpers et al., 2022 [102]
**Immunostaining-based assay**	Cytokeratin, cPARP, Ki67	Individual cells	No	Microscope	No	ICI efficacy can be evaluated	Kodack et al., 2017 [103] Collins et al., 2021 [104] Miles et al., 2021 [87]
**Immunostaining for drug** **specific response biomarkers**	RAD51 foci, γH2AX and p-c-Jun for DNA damaging agents. pS6 for MEK inhibitor.	Individual cells	No	microscope	No	Use small amount of ex vivo culture	Hill et al., 2018 [105] Compadre et al., 2023 [106] Tsukamoto et al., 2022 [107] Hirashita et al., 2021 [108]

^a^; Cells are lysed in bulk assays while each of the cells is evaluated in individual cell assays. ^b^; Most of the non-invasive assays can be serially performed over time. ^c^; Hallmark of an early apoptotic event.
